# Histopathology and indicators of borderline ovarian tumours with microinvasion in bitches

**DOI:** 10.17221/103/2022-VETMED

**Published:** 2023-04-12

**Authors:** Peter Makovicky, Peter Bolgac, Maria Makovicka, Karol Kajo, Zuzana Krchnikova, Jaroslav Pokoradi, Pavol Makovicky, Zuzana Stanova, Kristina Vaskova, Kvetoslava Rimarova

**Affiliations:** ^1^Infectious Diseases and Preventive Medicine, Veterinary Research Institute, Brno, Czech Republic; ^2^Department of Histology and Embryology, Faculty of Medicine, University of Ostrava, Ostrava, Czech Republic; ^3^Cancer Research Institute, Biomedical Research Centre of the Slovak Academy of Sciences, Bratislava, Slovak Republic; ^4^Department of Pathology, Veterinary and Food Institute, Bratislava, Slovak Republic; ^5^Department of Pathology, St. Elisabeth Cancer Institute, Bratislava, Slovak Republic; ^6^Veterinary Clinic – Animal Reproduction Centre, Budmerice, Slovak Republic; ^7^Department of Biology, Faculty of Education, J. Selye University, Komarno, Slovak Republic; ^8^Department of Zoology, Faculty of Natural Sciences, Comenius University, Bratislava, Slovak Republic; ^9^Department of Public Health and Hygiene, Faculty of Medicine, Pavol Jozef Safarik University in Kosice, Slovak Republic

**Keywords:** gynaecological diseases, implantation metastases, prognostic significance, veterinary pathology

## Abstract

The authors present two cases of borderline ovarian tumours with microinvasion in bitches with variable clinical significance. The first case documents a four-year-old female Weimaraner diagnosed with a tumour on the right ovary during a veterinary check-up, using ultrasound (USG) examination, which was then surgically removed. Histological examination revealed a clear cell borderline tumour of the ovary with microinvasion. The second case is represented by a necropsy sample from a 52-month-old female German Shepherd who died a day before the planned hysterectomy due to undertreated pyometra. During necropsy, a developed bilateral ovarian tumour was found. An additional histological examination revealed a serous borderline tumour with microinvasion of both ovaries. This paper discusses the histopathological and clinical aspects involved in the prognosis of borderline ovarian tumours in bitches. This concerns the possibility of a change for a more aggressive behaviour of these tumours and their immunohistochemical profile, then the risk of implant metastases and, finally, the time point of diagnosis, intervention, and therapy. Even histologically verified well-differentiated forms of borderline ovarian tumours with microinvasion in bitches can show the variable clinical significance and, therefore, in similar cases, only a good or only a bad prognosis of the disease should not be expected.

Canine ovarian tumours are less often diagnosed compared to other types of tumours, with one of the reasons being that many bitches are ovariectomised (Borzacchiello et al. 2010). They are mostly epithelial tumours of adult bitches, of which 30% to 50% are bilateral (Hori et al. 2006; Novotny et al. 2011; Rowan et al. 2017). Even macroscopically, they often have a typical cystic structure with numerous visible, thin, miniature intraluminal projections. Microscopically, they are characterised by a papillary arrangement with a rim of cubic or cylindrical cells. The benign form of papillary and cystic tumours is not associated with diagnostic difficulties. Malignant forms are microscopically characterised by an irregular papillary, cystic structure, disorganisation of glandular structures, cellular and nuclear atypia, increased mitotic activity, and stromal infiltration (Yotov et al. 2005). Clear cell tumour (CCT) of the ovaries is a less frequent finding compared to serous carcinoma and granulosa cell tumour (GCT) of the ovaries (Banco et al. 2011). CCT often has a cystic structure arranged in a tubular, papillary, but also solid pattern. Compared to serous carcinoma, however, its papillae are less hierarchical, while the cells are larger, cytoplasmically pale, and more atypical in appearance. The human histopathological practice has shown that, in addition to malignant forms of these tumours, there is also a group of borderline or intermediate tumours, which microscopically have some signs of carcinoma, but at the same time appear benign. Due to their appearance, they could be included in the group of tumours with higher prognostic significance compared to malignant forms of these tumours. In this sense, the finding can also be accompanied by microinvasion. According to the current veterinary pathology classification of ovarian tumours, there is no mention of the borderline ovarian tumour in bitches. On the other hand, our veterinary biopsy practice documented that ovarian tumours often show differences in histology and it is still questionable if it has an impact on prognostic significance. In the available veterinary literature, only sporadic data on the histopathology and indicators of such tumours in bitches can be found. Based on the hypothesis that this is not a homogeneous group, but tumours with different clinical courses, the objective of our study is to draw attention to this group of tumours with detailed histological descriptions. This paper is based on two cases of different ovarian tumours. Their histopathological views and indicators are documented. The variability of the prognostic significance of borderline ovarian tumours and also the importance of this diagnosis for veterinary biopsy practice are discussed.

## Case description

### CASE 1

**Figure 1:**
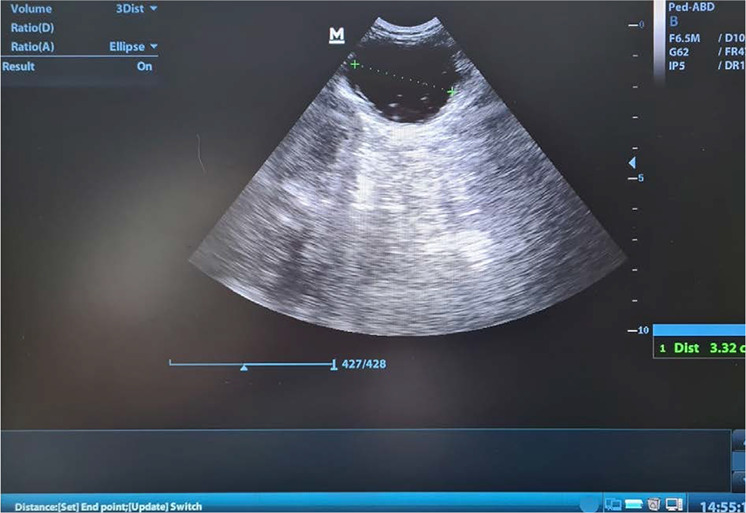
USG image showing a 3.32 cm wide tumour on the right ovary

**Figure 2:**
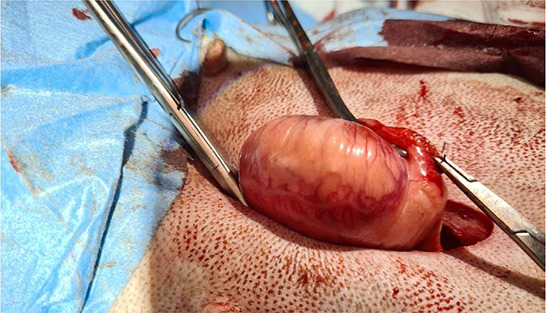
A view of the ovary during ovariectomy

**Figure 3:**
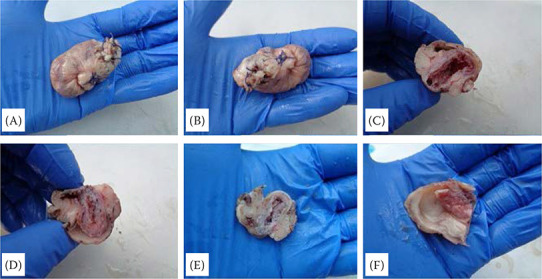
Ovary with several well-holding sutures (A, B) and, after cross-section, an optically empty cavity with somewhat free clear fluid (C, F) and, in one place on one side, a tumour attached to the wall, which almost completely fills the cavity (D) and from which three samples were taken (E)

A four-year-old Weimaraner female was examined in a veterinary clinic for a preventive check-up. A year ago, she suffered an abortion with subsequent endometritis, which was treated with antibiotics. *Klebsiella oxytoca* and *Mycoplasma* spp. were isolated from the aborted foetus and vaginal discharge at that time, using quantitative Real-Time PCR detection of DNA/RNA of the microorganisms (PregnancyMultiPlex_DOG test). Subsequently, the owner did not report any pathological changes. During the ultrasonography (USG) examination, an ovoid tumour-like mass filled with free fluid was seen on the right ovary, which was 3.32 cm in diameter (Figure 1). Unilateral ovariectomy with postoperative therapy followed, and after discharge from the hospital, the bitch was in home care on antibiotic treatment (Figure 2). The removed ovary was sent for histopathological examination and the collected fluid for bacteriological examination with the finding of *Staphylococcus* spp., *Streptococcus* spp., and *Escherichia coli* (PregnancyMultiPlex_DOG test). The entire ovary measuring 10 × 5 × 5 cm was delivered to the histopathology laboratory (Figure 3A,B). After cutting the ovary, there was a small amount of clear fluid. In the centre of the ovary, a yellowish formation, approximately in the shape of a pigeon egg, was visible, which was partially attached to the adjacent ligamentous capsule and in one place conspicuously merged with the wall of the capsule (Figure 3C–E). In addition, the ovary was formed by another cavity without tissue content (Figure 3F). A total of three samples were taken for histopathological examination.

### CASE 2

**Figure 4:**
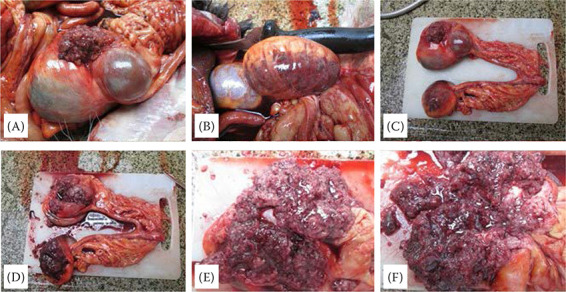
During the autopsy, both ovaries were visibly enlarged, with tumorous changes (A, B, C) and in one place also with tumour growth through the capsule with massive cauliflower-like formations in the place of both ovaries (D, E, F)

**Figure 5:**
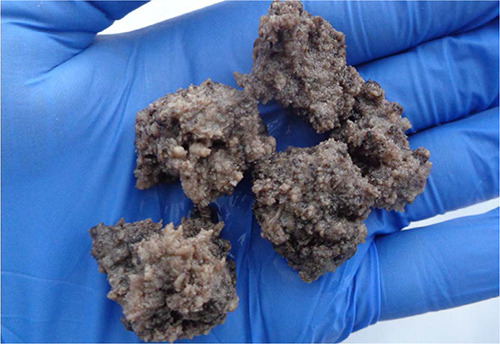
Five amorphous samples delivered to the laboratory, with numerous fine surface protrusions that easily broke off and crumbled into miniature pieces

This was a female German Shepherd aged 52 months. During the veterinary examination, the owner reported an excessively long oestrus alternating with a quiet period without heat. The examination diagnosed pyometra, which was successfully cured with medication. Subsequently, the bitch did not show problems for seven months and then pyometra was diagnosed again. Antibiotic therapy only partially improved the patient’s health condition. The signs persisted with fluctuating temperature, loss of appetite, and increased sensitivity to touch in the pelvic and abdominal regions. Gradually, the bitch became more apathetic, which was accompanied by progressing inappetence and wasting away. Another professional examination, using USG, revealed tumorous changes in both ovaries. In agreement with the owner, a hysterectomy was to be performed the following day. However, in the morning, the owner reported the death of the patient. He decided on a necropsy, in which changes in the size and shape of the ovaries were detected (Figure 4A,B). These were partially covered by a thin superficial fibrous capsule with variably sized cavities distended by fluid (Figure 4C). In one place, it grew through as a superficial capsule cauliflower-like tumorous formation. After cutting the superficial capsule, there were visible bilateral cauliflower-like red to red-brown coloured tumours which completely filled almost the entire ovaries (Figure 4D). After cutting the tumours, blood-stained cystic cavities appeared bilaterally with fine dark projections (Figure 4E,F). The uterus was haemorrhagically infiltrated and, after cutting, some oozing pale secretion viscous to the touch was observed. Other internal organs were without macroscopically visible changes. A total of five samples were taken from different parts of both tumorous ovaries (Figure 5).

## Histochemical and immunohistochemical assays

After collection, the tumour tissue samples were fixed in a solution of 10% formalin for 24 hours. The material was excised and subsequently processed by a standard histological embedding technique into paraffin blocks. The blocks were cut on a Hyrax M40 rotary microtome (Zeiss, Jena, Germany) and tissue sections were placed on specialised silanized Star Frost^®^ glass slides (Waldemar Knittel, Brunschweig, Germany). The first series of sections were stained with standard haematoxylin-eosin staining (H&E) (Bamed, České Budějovice, Czech Republic). A second series of sections were stained for evidence of collagen, using the Masson trichrome histochemical kit (Bamed, České Budějovice, Czech Republic). Another series of sections were processed using special immunohistochemical techniques with evidence of several antibodies (Dako Omnis, Copenhagen, Denmark). These are Alpha-1-Fetoprotein Antibodies (AFP), CD68 Antibodies (CD68), Estrogen Receptor α Antibodies (ER), Inhibin α Antibodies (Inhibin), Ki-67 Antigen Antibodies (Ki-67), p-53 Protein Antibody (p-53), and finally Wilms Tumour 1 Protein Antibody (WT1). Before immunostaining, heat-induced antigen retrieval was performed by a 20 min treatment in a PT Link (Dako Omnis, Copenhagen, Denmark), using pH 9.0 buffer (EnVision Flex Target Retrieval Solution, High pH; Dako Omnis, Copenhagen, Denmark). After this, the slices were allowed to cool and then incubated with antibodies for 30 minutes at room temperature. For the washing of slices, a conventional wash buffer was used (EnVision Flex Wash Buffer; Dako Omnis, Copenhagen, Denmark). For visualization, an LSAB2 System-HRP (Dako Omnis, Copenhagen, Denmark) was applied according to instructions. The reaction was visualized with EnVision Flex DAB+chromogen (Dako Omnis, Copenhagen, Denmark). Finally, the slices were stained with Mayer haematoxylin (Bamed, České Budějovice, Czech Republic). The samples were described and evaluated in a light-microscopic picture, using an optical microscope (Axiovert 200; Zeiss, Jena, Germany).

## RESULTS

### Case 1

**Figure 6:**
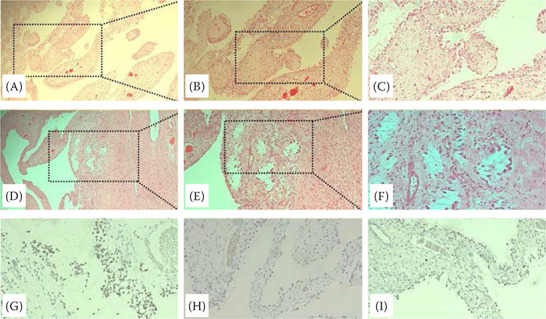
View into the centre of the tumour, which is formed by a cystic cavity with several blunt pseudopapillary formations growing into the lumen, which are lined with multilayered epithelium (A–C) In the stroma of the medulla, several miniature pseudofollicular tumorous formations are present (D–F). Immunohistochemical positivity for ER (G), negativity of CD68 (H) and individual positivity for Ki-67 (I). (A,D) H&E × 5; (B,E) H&E × 10; (C,F) H&E × 20; (G) ER: H × 20, (H) CD68: × 20; (I) Ki-67: × 20

Histologically, it was a clear cell borderline tumour with microinvasion of the ovary, which was formed by a centrally located cystic cavity with several blunt pseudopapillary formations, which were predominantly normal in shape and appearance, partly also branching more extensively, growing into the lumen (Figure 6A–C). The cystic protrusions were covered by a multi-layered epithelium with the presence of cells with voluminous pale to completely transparent cytoplasm and one ovoid protrusion, with a darker core. Some of the formations were found to be freely floating in the lumen of the cystic cavity in the form of several isolated, variably sized clumps of tissue. There were several tumorous formations in the deeper parts of the ovary microformations that were formed by microglands, thus imitating empty follicles with a rim of a multi-layered epithelium with the presence of apparently irregular, slightly voluminous cells with one formation with a darker core (Figure 6D–F). Immunohistochemically, ER expression was demonstrated in less than 50% of tumour cells (Figure 6G); inhibin and CD68 were negative (Figure 6H). Proliferative activity assessed by Ki-67 was noted in approximately 10% of tumour cells (Figure 6I) and p53 showed wild-type expression.

### Case 2

**Figure 7:**
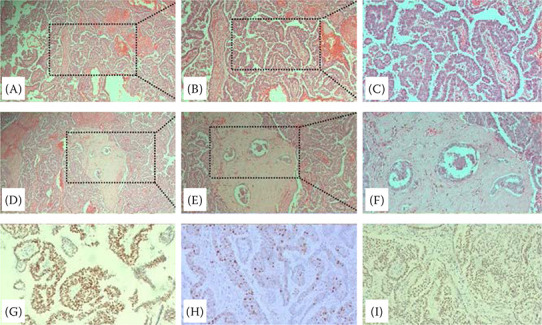
A tumour that is composed of numerous thin papillary formations (A–C) with stromal infiltration (D–F) and immunohistochemically positive reaction for ER (G), positive reaction for Ki-67 (H) and positive reaction for WT1 (I) (A,D) H&E × 5; (B,E) H&E × 10; (C,F) H&E × 20; (G) ER: × 20, (H) Ki-67: × 20, (I) WT1: × 20

Histologically, there was a serous borderline tumour of both ovaries with microinvasion. The tumour was composed of densely packed and partially compressed, richly branched, mostly formed, thin papillary formations, which were lined by relatively uniform cells containing a single ovoid vitreous nucleus with a minimum of clumped chromatin (Figure 7A–C). Masson trichrome staining showed positive areas with a well-vascularized stroma and several isolated tumorous microglands, which represented areas of microinvasion (Figure 7D–F). A smaller part of the tumour was subject to necrosis. The overall histological picture was completed by an isolated round-cell inflammatory infiltrate. Immunohistochemically, diffuse WT1 positivity (Figure 7I) and ER expression (up to 60% of tumour cells) were demonstrated in tumour cells (Figure 7G). AFP evidence was negative. Proliferative activity assessed by the Ki-67 index reached 40% (Figure 7H).

## DISCUSSION AND CONCLUSION

Despite the lower incidence of ovarian tumours in dogs, this issue is intensively discussed in the professional veterinary literature, with cases from practice predominating. According to the international veterinary classification, four groups of ovarian tumours of dogs are defined. Tumours arising from the surface coelomic epithelium (TSCE), then sex cord-stromal tumours (SCST), germ cells tumours, and, finally, mesenchymal tumours (Kennedy et al. 1998). The mentioned categories include several types of tumours. There are certain differences in the professional literature regarding the frequency of ovarian tumours in bitches. According to Maxie (2016), Lee et al. (2007) the most common TSCEs are papillary cystadenoma and papillary adenocarcinoma. Other sources state that the majority of findings in bitches are GCTs, which are included in the SCST group (Heil da Silva Matos et al. 2021). For example, in one retrospective histopathological study based on 71 cases of female ovarian tumours, TSCEs accounted for 46%, SCSTs for 34%, and germ cell tumours for 20% of all tumours (Patnaik and Greenlee 1987). From the point of view of prognosis, possible differences in the survival time of dogs with individual types of ovarian tumours are particularly noteworthy. The results of one study showed that bitches with GCT had a longer median survival time (822–1 840 days) compared to median survival time in bitches with malignant epithelial tumours (617–841 days) (Itoh et al. 2021). Similarly, according to the results of another study, bitches with GCT show a longer average survival time (1 474 days) compared to bitches diagnosed with adenocarcinoma and dysgerminoma (458 days) (Goto et al. 2021). Similarly to serous papillary tumour, we diagnosed CCT based on its appearance in the clear colour image. Microinvasiveness was determined by the presence of tumour cells in the stroma, and the borderline nature of the tumours was determined based on their proliferative appearance. Both types of tumours were subsequently subjected to special immunohistochemical examinations. Another study reported that ovarian adenocarcinomas are often accompanied by ascites, which includes a positive reaction with WT1 and then also with progesterone receptors (PR) (Kita et al. 2022). In the case of the borderline serous tumour with microinvasion, we recorded an almost diffuse positivity of WT1, which is positive in cases of TSCE (Riccardi et al. 2007). Within the hormonal profile, we preferentially chose ER because PR are subordinate to them, but on the other hand, both are commonly present in other types of ovarian tumours. This is also evidenced by our results, in which both types of tumours were shown to be endocrine active with a positive ER reaction. Although we could have diagnosed both cases based on H&E staining, in our opinion, the special immunohistochemical examinations performed are relevant. In certain circumstances, there may be difficulties in diagnosing these tumours (Pichon et al. 2011). We found sporadic information in the professional literature about borderline ovarian tumours in bitches. At the same time, these tumours are diagnosed in human medicine in up to 20% of all epithelial ovarian tumours (Carbonnel et al. 2021; Lawton and Pavlik 2022). Therefore, it is likely that they will also occur in bitches to some extent. Perhaps one of the first cases involving the finding of microinvasion was described in a 6-year-old female Pointer dog (Demirel and Ergin 2016). The histological findings can be compared with our case of serous borderline tumour with microinvasion, which, however, we expanded with selected immunohistochemical examinations. In another study, there is a histological description of a disseminated low grade serous adenocarcinoma of the ovaries of a badger (*Meles meles* L.), which the authors compare to human borderline ovarian tumours with evidence of WT1 and Ki-67 (Kutlvasr et al. 2014). This case can also be similarly compared with our histological findings. Here, on the basis of our second case, we document that despite the well-differentiated form of an exophytically growing serous borderline tumour with microinvasion, only a good prognosis cannot be expected. Even though pyometra was established as the primary cause of death in this case, the tumour showed bilateral intensive growth with worse prognosis. On the contrary, in the first case, we presented a well-differentiated cystic endophytically growing borderline CCT with microinvasion, and no recurrence was noted after unilateral ovariectomy. Thus, it is possible that prognostic significance may depend on three factors. The first factor is, despite the intermediate appearance of these tumours, the possibility of a change to a potentially more aggressive form of behaviour. A wider immunohistochemical panel will report on these facts. For example, Ki-67, which we chose, showed differences in the proliferative activity of tumour cells between two tumours, while we recorded a lower mitotic index in the case of CCT. However, these parameters should also be evaluated in the context of the histological findings, because a higher proliferation index is a worse sign in the case of an endophytically growing tumour compared to an exophytically growing tumour. The multifunctional protein p53 functions as a transcription factor, with the ability to control the functionality of individual genes, including the ability to suppress DNA replication and the ability to regulate apoptosis (Hernandez-Suarez et al. 2022). Therefore, it can be concluded that the identification of the accumulation of standard and mutated forms of p53 is one of the alternative additional examinations with prognostic significance of ovarian tumours in bitches. We evaluated several architecturally complex formations of tumorous cells in the stroma of the ovary as microinvasive growth. For these types of tumours, borderline forms have not yet been defined in veterinary pathology, but both of our cases could be examples. The second factor is the risk of implantation metastases, because TSCEs can outgrow the surface cases of the ovaries, while the vital parts of the tumour easily fall into the surrounding environment and get caught on the wall of the peritoneum. Then they can invade here the wall and establish new deposits in several places. Also, in the second case we presented, one of the ovaries was macroscopically clearly overgrown by a tumour, while the tumour easily disintegrated or even crumbled into miniature parts during the excision process. Although no implant metastases were macroscopically noted at autopsy, it is possible that the tumour itself had already spread in this way. Conversely, the finding of microinvasiveness without desmoplasia in the form of several miniature tumourous glands may not be manifested by the establishment of new foci, because it is most likely a local spread of the tumour. This is evidenced by our first case, where no secondary tumour foci were noted even after ovariectomy, but also by our second case without pathology on other organs and without clearly macroscopically visible secondary tumour foci in other organs. The third factor is the time point of view: in the second case, the tumour was diagnosed in a significantly developed form compared to the first case. Although it is clear that the temporal aspect is of prognostic importance, our cases do not clearly document this. As mentioned in the methodological section, in both cases, gynaecological problems were first recorded, which were managed therapeutically, and only then were ovarian tumours diagnosed. It follows that even after a positive response to therapy, bitches with repeated gynaecological problems should continue to be checked by a veterinarian on an outpatient basis at regular intervals.

Here, on the basis of two cases from practice, we document the different microscopic, macroscopic appearance and clinical behaviour of two ovarian tumours in bitches, with the recommendation to distinguish between their intermediate forms, i.e. borderline tumours. It is clear that even histologically well-differentiated forms of ovarian tumours can behave differently. Microinvasion without desmoplasia does not appear to be risky. The prognosis itself is rather dependent on the way the tumour grows, the appearance of the cells and the proliferation index of the tumour cells. Furthermore, implantation metastases, metastases in other organs, then the time of diagnosis, surgery and finally therapeutic aspects affect their prognosis.
